# Evolution, Expression, and Function of Nonneuronal Ligand-Gated Chloride Channels in *Drosophila melanogaster*

**DOI:** 10.1534/g3.116.029546

**Published:** 2016-05-04

**Authors:** Emily J. Remnant, Adam Williams, Chris Lumb, Ying Ting Yang, Janice Chan, Sebastian Duchêne, Phillip J. Daborn, Philip Batterham, Trent Perry

**Affiliations:** * School of BioSciences, University of Melbourne, Victoria 3010, Australia; † Bio21 Molecular Science and Biotechnology Institute, University of Melbourne, Victoria 3010, Australia; ‡ Behaviour and Genetics of Social Insects Laboratory, School of Life and Environmental Sciences, University of Sydney, New South Wales 2006, Australia; § Charles Perkins Centre, School of Life and Environmental Sciences, University of Sydney, New South Wales 2006, Australia

**Keywords:** ligand-gated chloride channel, gene duplication, copy number variation, nonneuronal expression, copper tolerance

## Abstract

Ligand-gated chloride channels have established roles in inhibitory neurotransmission in the nervous systems of vertebrates and invertebrates. Paradoxically, expression databases in *Drosophila melanogaster* have revealed that three uncharacterized ligand-gated chloride channel subunits, CG7589, CG6927, and CG11340, are highly expressed in nonneuronal tissues. Furthermore, subunit copy number varies between insects, with some orders containing one ortholog, whereas other lineages exhibit copy number increases. Here, we show that the Dipteran lineage has undergone two gene duplications followed by expression-based functional differentiation. We used promoter-GFP expression analysis, RNA-sequencing, and *in situ* hybridization to examine cell type and tissue-specific localization of the three *D. melanogaster* subunits. CG6927 is expressed in the nurse cells of the ovaries. CG7589 is expressed in multiple tissues including the salivary gland, ejaculatory duct, malpighian tubules, and early midgut. CG11340 is found in malpighian tubules and the copper cell region of the midgut. Overexpression of CG11340 increased sensitivity to dietary copper, and RNAi and ends-out knockout of CG11340 resulted in copper tolerance, providing evidence for a specific nonneuronal role for this subunit in *D. melanogaster*. Ligand-gated chloride channels are important insecticide targets and here we highlight copy number and functional divergence in insect lineages, raising the potential that order-specific receptors could be isolated within an effective class of insecticide targets.

Ligand-gated ion channels (LGICs) are derived from an ancient family with prokaryotic LGIC-like homologs thought to function as chemoreceptors (Tasneem *et al.* 2004). They mediate fast ionotropic synaptic signaling in the central and peripheral nervous systems of vertebrate and invertebrate organisms. LGICs are transmembrane receptors comprised of five subunits that facilitate the passage of ions through a central pore upon activation with a ligand. They consist of a large, extracellular, N-terminal region containing ligand binding domains, four transmembrane (TM) domains that line the ion channel pore, and an intracellular loop between TM3–4. There are two monophyletic branches: cationic excitatory receptors gated by the neurotransmitters serotonin and acetylcholine, and inhibitory ligand-gated chloride channels (LGCCs), gated by GABA, glycine, histamine, and glutamate (Cully *et al.* 1994; Ortells and Lunt 1995; Raymond and Sattelle 2002; Liu *et al.* 2005; Dent 2006; Absalom *et al.* 2009). The metazoan LGIC lineages expanded from clades present in the last common bilaterian ancestor, and extant metazoa now exhibit lineage-specific clades (Dent 2006). Nine invertebrate-specific LGCC classes have been identified, including the histamine- and glutamate-gated chloride channels (Cully *et al.* 1994; Raymond and Sattelle 2002; Liu *et al.* 2005; Dent 2006).

The *Drosophila melanogaster* genome contains 12 LGCC subunit genes (Supplemental Material, Figure S1). Although the subunit complement is smaller than the number present in the nematode and mammalian genomes (∼35 LGCCs in *Caenorhabditis elegans*; ∼23 in *Rattus norvegicus*; Dent 2006; Jones and Sattelle 2008), the insect LGCC subunits show diversity in conserved ligand binding and TM domains (Knipple and Soderlund 2010), suggesting that the small number of subunits have a wide range of activating ligands and physiological functions. At least four *D. melanogaster* LGCC subunits produce receptors that are targets for insecticide binding. The *Rdl* *(Resistance to Dieldrin)* GABA receptor is a target site for cyclodienes, phenylpyrazoles, and isoxazolines (Cole *et al.* 1993; Ozoe *et al.* 2010; Remnant *et al.* 2014), and the glutamate-gated receptor subunit *GluClα* and histamine receptor subunits *HisCl1* and *ort* are associated with avermectins (Cully *et al.* 1996; Iovchev *et al.* 2002; Yusein *et al.* 2008). Although the role of LGCC subunits as insecticide targets has been intensely studied, the endogenous functions of LGCCs in *D. melanogaster* continue to be under investigation. Thus far, roles for the histamine receptors *HisCl1* and *ort* in the visual system have been established (Gisselmann *et al.* 2002; Witte *et al.* 2002; Zheng *et al.* 2002); *Rdl* is involved in olfactory memory (Liu *et al.* 2007), sleep (Agosto *et al.* 2008; Chung *et al.* 2009), and male aggression (Yuan *et al.* 2014); *GluClα* is involved in circadian rest and arousal (McCarthy *et al.* 2011) and light avoidance (Collins *et al.* 2012); and *pHCl* was identified as being gated by changes in pH (Schnizler *et al.* 2005). *Lcch3* and *Grd* form functional GABA-gated cation receptors *in vitro*, (Gisselmann *et al.* 2004) and *Lcch3* has a potential role with *Rdl* in olfaction (Dupuis *et al.* 2010), whereas *Grd* has an unknown function. The remaining five subunits (CG8916, CG12344, CG11340, CG7589, and CG6927) are thus far uncharacterized (Jones *et al.* 2010; Knipple and Soderlund 2010).

Most insect genomes contain 10–12 LGCC subunits (Jones and Sattelle 2006, 2007; Dale *et al.* 2010; Jones *et al.* 2010). In holometabolous insects, LGCC subunits are highly conserved and orthology can be clearly determined (Figure S1), suggesting that the basic subunit complement was present in early common ancestors of this lineage. One-to-one orthology diminishes in the Hemipteran aphid *Acyrthosiphon pisum* genome, with five subunits containing no direct orthologs (Dale *et al.* 2010; Figure S1). Even within the highly conserved LGCC subunits of the holometabola, there are lineages that display divergent gene copy number. Copy number variation (CNV) has been identified in the insecticide target subunit *Rdl*, in the Lepidoptera (three subunits; Yu *et al.* 2010), and in aphids (two subunits; Anthony *et al.* 1998; Dale *et al.* 2010). Recently in *D. melanogaster*, ancestrally single copy for *Rdl* lines have been isolated with an *Rdl* duplication, containing key mutations involved in insecticide resistance (Remnant *et al.* 2013), suggesting that CNV in the *Rdl* insecticide target may provide a selective advantage under multiple environmental conditions.

A second LGCC clade, referred to as the Insect Group I subunits (Dent 2006), have no known ligands or functions (Knipple and Soderlund 2010) and exhibit CNV in insect species (Jones and Sattelle 2006, 2007). Online expression databases evoke further ambiguity about the three *D. melanogaster* Insect Group I subunits (referred to hereafter as CG7589, CG6927, and CG11340; Figure S2). Genome-wide spatial and temporal expression information (available at http://flybase.org/) from FlyAtlas (Chintapalli *et al.* 2007) and ModEncode (The modENCODE Consortium *et al.* 2010) indicates that CG7589, CG6927, and CG11340 show remarkably different expression patterns in nonneuronal tissues, in contrast to the remaining nine LGCC subunits that are enriched in the larval and adult central nervous system, thoracoabdominal, ganglion and head, expression patterns typical of genes predicted to be involved in fast synaptic transmission and neuronal processes characteristic of LGCCs (Figure S2). The unique and divergent expression patterns of the Insect Group I LGCC subunits, and the propensity for CNV in insect species, necessitates further investigation into their function.

Here, we explore the evolution, expression, and function of *D. melanogaster* LGCC Insect Group I subunits. We explore CNV, examining the phylogenetic relationship of Insect Group I LGCC orthologs from sequenced insect species. We describe the expression of *D. melanogaster* CG7589, CG6927, and CG11340, illustrating specific tissue and cell type expression patterns. Finally, we investigate the function of CG11340 using a genomic knockout. Understanding the functional repertoire of divergent receptor subunits within the LGCC family has the potential to reveal pest-specific target sites where orthologs are present only in pests that show sufficient divergence from nontarget insects.

## Materials and Methods

### Identifying Insect Group I LGCCs and phylogenetic analysis

We identified orthologous Insect Group I subunits by tBLASTn searches with amino acid sequences of CG6927, CG7589, and CG11340 against a selection of sequenced insect genomes. Reciprocal BLAST searches back to the *D. melanogaster* genome were conducted to determine one-to-one orthology. The subsequent top hits from BLAST searches of insect genomes were also checked against the *D. melanogaster* genome to ensure that a different member of the LGCC family was the true ortholog. Where required, annotations were performed manually or existing annotations were modified in Artemis. Identified orthologs were combined with previously published results (Jones and Sattelle 2006, 2007; Dale *et al.* 2010; Jones *et al.* 2010) to generate the Insect Group I alignment.

We aligned coding regions using Muscle v3.8 (Edgar 2004) and visually inspected the alignment. We then estimated a phylogenetic tree using maximum likelihood in RaxML. We used the GTR+G substitution model, which had the optimal BIC score among the set of substitution models compared in jModelTest 2 (Darriba *et al.* 2012). We assessed branch support by conducting 1000 bootstrap replicates under the same settings.

### Generating 5′ promoter-GFP constructs for CG7589 and CG6927

Primers were designed to amplify the 5′ upstream sequences of CG6927 (1.7 kb) and CG7589 (3.9 kb) from *w^1118^* genomic DNA (Figure 2). The 5′ fragments incorporated the intervening genomic sequence commencing from the end of the nearest upstream gene to the start codon of each subunit (Figure S4). The 5′ promoter constructs were cloned into pGEM, sequenced, and then subcloned into the pStinger vector (Barolo *et al.* 2000), directly upstream of a nuclear GFP open reading frame, and pC3G4 vector (Thummel and Pirrotta 1991), upstream of a GAL4 open reading frame, to generate GFP and GAL4 driver lines, respectively. Microinjection of pStinger and pC3G4 plasmids (300 ng/μl, and 100 ng/μl Δ2-3 transposase helper plasmid, following standard microinjection protocols (Rubin and Spradling 1982) using a Femtojet (Eppendorf) and FemtotipII needles. Emerging adults were backcrossed to w1118 and offspring with w^+^ red-eye phenotype indicating integration events were balanced using *If*/CyO; TM6b/MKRS balancer stock, and made homozygous or maintained over balancers where required.

### Generation of GFP-CG11340-P[acman] construct

BACPACs were ordered (DGRC), homology arms designed, and recombineering performed as per Venken *et al.* (2006). The modified BAC fused the GAL4 coding region, in-frame to the start codon and was predicted to express Gal4 in a pattern controlled by elements that regulate the native expression of the specific gene. Microinjection as per above into the 51C strain led to integration at the attP landing site on chromosome 2, which was removed via flanking Lox sites using crosses to flies that express Cre-recombinase (BDSC#1092). The resulting expression patterns were visualized by crossing males to UAS > GFP.nls lines (BDSC#4775).

### GFP expression and imaging

At least five independent insertion lines for pStinger 5′ promoter constructs were observed for GFP expression pattern for each gene. A similar number of independent lines containing the pC3G4 promoter-GAL4 constructs for each gene were crossed to UAS-nGFP and UAS-mGFP lines and observed for GFP expression. Larval and adult tissues were dissected in PBS and expression was imaged using a UV filter on the SZX12 stereomicroscope system (Olympus).

### Overexpression constructs

The open reading frames of CG6927 (1.86 kb), CG7589 (1.84 kb), and CG11340 (1.61 kb) were amplified from w^1118^ cDNA, generated with RNA extracted from ovaries for CG6927, and third instar larvae for CG7589 and CG11340. Primers were designed to amplify from the 5′ untranslated region to the stop codon and subcloned into the pUAS-attB vector (Bischof *et al.* 2007). Plasmids were microinjected into two *attP* strains, 51C (chrII) and 86Fb (chrIII), as above, with successful integration events confirmed as homozygous by PCR.

### In situ hybridization

*In situ* hybridization of adult female ovaries was carried out using a modified protocol from Tautz and Pfeifle (1989). Primers were designed to amplify a 1067 bp fragment from the coding sequence of CG6927. The fragment was cloned in both the forward (sense probe) and reverse (antisense probe) directions into pGEM, and checked by sequencing. Plasmid DNA was purified using a Qiagen MIDI-prep kit. To create the sense (control) and antisense probes, 10 μg of each plasmid was linearized at the 3′ end of the insert by overnight restriction digest with *Sal*I, purified by ethanol precipitation, and transcribed at 37° for 2 hr using Megascript T7 RNA polymerase (Ambion), incorporating DIG-labeled nucleotides.

One-day-old wild-type adult females were placed with males onto standard fly media containing an active yeast paste and left for 3 d to ensure mating. Three replicates of 5–10 ovaries were dissected from 4-day-old mated females in PBS and placed in Eppendorf tubes. Tissues were fixed with 4% paraformaldahyde, washed, and prehybridized, before incubating with the antisense or sense probe at 55° overnight. Postincubation, ovaries were washed and incubated with 1:1000 Anti-Digoxigenin-AB, Fab fragments (Roche) for 2 hr, followed by a developing reaction with 1:50 NBT/BCIP solution (Roche) for 15–20 min. Ovaries were mounted in 80% glycerol, and imaged using the SZX12 stereomicroscope system (Olympus).

### Generating CG11340 knockout

#### Cloning of the pGX donor construct:

Deletion of the genomic region containing the CG11340 coding sequence was conducted using the ends-out method (Gong and Golic 2003; Huang *et al.* 2008). Primers introduced *Kpn*I and *Spe*I restriction sites, respectively, to 5023 bp 5′ and 2986 bp 3′ homology arms using PCR and sequential cloning into the pGX-attP vector (pGX-attP^CG11340^).

#### Creation of donor D. melanogaster lines:

Microinjection of pGX-attP^CG11340^ (300 ng/μl) and Δ2-3 transposase plasmid (250 ng/μl) into w^1118^ followed the injection, integration, and crossing procedures described above. The four most efficient second chromosome transgenic donor lines for UAS-Reaper efficiency with *GAL4^447w^*^-^ were used for the ends-out crossing scheme (Huang *et al.* 2008). Donor lines crossed with the *6934-hid* strain produced mosaic F1 females that were then crossed to *GAL4^447w^*^-^ males to remove lines that had retained the pGX vector containing UAS-Reaper. Candidate lines from this with solid eye color were collected and mapped to chromosomes by crossing to *Dba*l, with those mapped to the incorrect chromosome discarded leaving four ends-out candidate lines. Southern blot experiments confirmed that the genomic region was deleted for these lines (Figure S5), and RT-PCR of CG11340 illustrated that no transcript was produced in deletion mutants (data not shown). The ends-out construct was removed with cre-recombinase by crossing knockouts to Cre-DBal flies to leave the deleted region of CG11340 and an attP landing site.

### Bioassays

Larval toxicity assays in this study for analysis of 5′HR driving tissue-specific overexpression and RNAi of CG11340 and CG7389 in the midgut, malpighian tubules, and fat body, and of the ends-out knockout lines, were performed as per Perry *et al.* (2012), using four to five replicates of 50 first instar larvae placed onto standard fly media containing either CuSO4, CuCl_2_, the copper chelator BCS, ZnCl_2_, ZnSO_4_, or H_2_O (control). Survival to adulthood (eclosion) was scored, and mortality data corrected for control mortality using Abbott’s correction (Hoekstra 1987; Rosenheim and Hoy 1989), with 95% confidence interval calculations.

### Reagent and data availability

Strains generated in this study are available on request. Sequences used in the LGCC Insect Group 1 alignment are provided in File S1.

## Results

### Insect Group I LGCC subunit CNV

**Figure 1:**
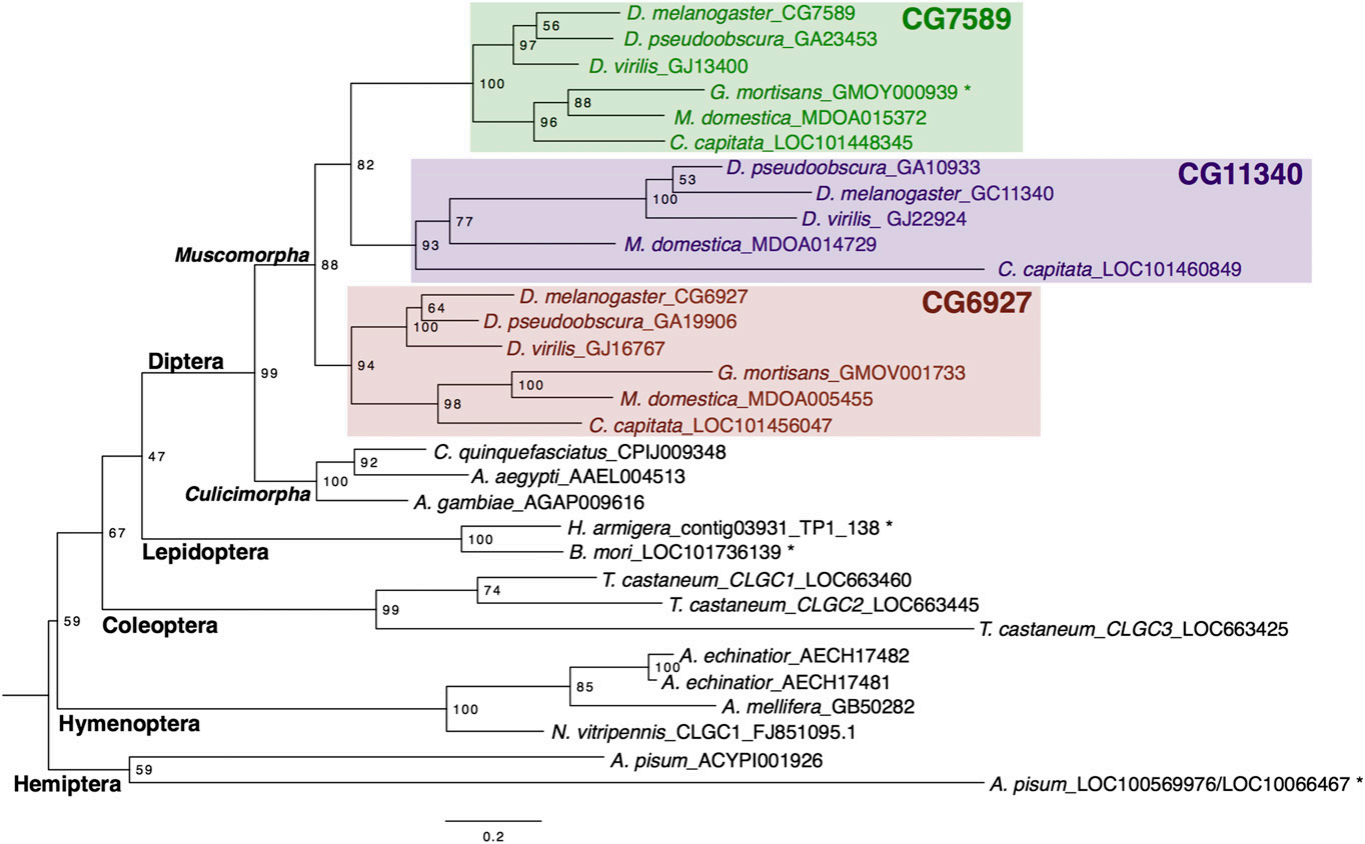
Maximum likelihood phylogenetic tree of Insect Group I LGCCs. Node labels indicate branch support as a percentage over 1000 bootstrap replicates. The scale bar denotes the expected number of substitutions per site along branches in the tree. * indicates manually annotated or corrected.

Phylogenetic analysis of orthologous Insect Group I LGCC subunits indicated that copy number varies across the insect species examined (Figure 1 and Figure S1). The aphid *A. pisum* contains two Insect Group I-like subunits (Dale *et al.* 2010), and the red flour beetle *Tribolium castaneum* contains three subunits (Jones and Sattelle 2007). In both species, the subunits are monophyletic. In the Hymenopteran species examined, *Apis mellifera* and *Nasonia vitripennis* contain one Insect Group I subunit (Jones and Sattelle 2006; Jones *et al.* 2010), whereas a third Hymenopteran, the ant *Acromyrmex echinator*, contains two subunits tandemly arranged on the same scaffold (Figure S3). Both Lepidopteran species examined, *Bombyx mori* and *Helicoverpa armigera*, have a single Insect Group I ortholog. In the Dipteran lineage, all three mosquitos examined (*Aedes aegypti*, *Culex pipiens*, and *Anopheles gambiae*) have a single ortholog. However, in the Muscomorpha, most species examined contain three paralogous subunits (*D. melanogaster*, *D. pseudoobscura*, *D. virilis*, *Musca domestica*, and *Ceratitis capitata*). *Glossinia mortisans* is the only species in this clade that contains two rather than three subunits, missing a CG11340 ortholog (Figure 1). We examined intron–exon structure and saw that, in *D. melanogaster* and *G. mortisans*, CG7589 contains seven exons and a number of splice boundaries shared with the single *Ap. mellifera* and *Ae. aegypti* orthologs (Figure S3).

### Distinct expression patterns in peripheral tissues

To explore expression patterns, we examined nuclear and membrane-bound GFP expression for multiple constructs for each LGCC subunit in dissected tissues from adults and wandering third instar larvae. For CG7589 and CG6927, we cloned the 5′ upstream sequence of each subunit into two expression vectors: pStinger, directly in front of a green fluorescent protein (GFP) open reading frame, and pC3G4, driving expression of GAL4, which could subsequently be crossed to different UAS-GFP lines for GFP expression (Thummel and Pirrotta 1991; Brand and Perrimon 1993; Barolo *et al.* 2000; Figure S4). For CG11340, we used a recombineering approach to insert a GAL4 open reading frame at the start codon of CG11340 inserted into a P[acman] clone (Venken *et al.* 2006) containing the full length genomic sequence.

#### CG7589:

**Figure 2:**
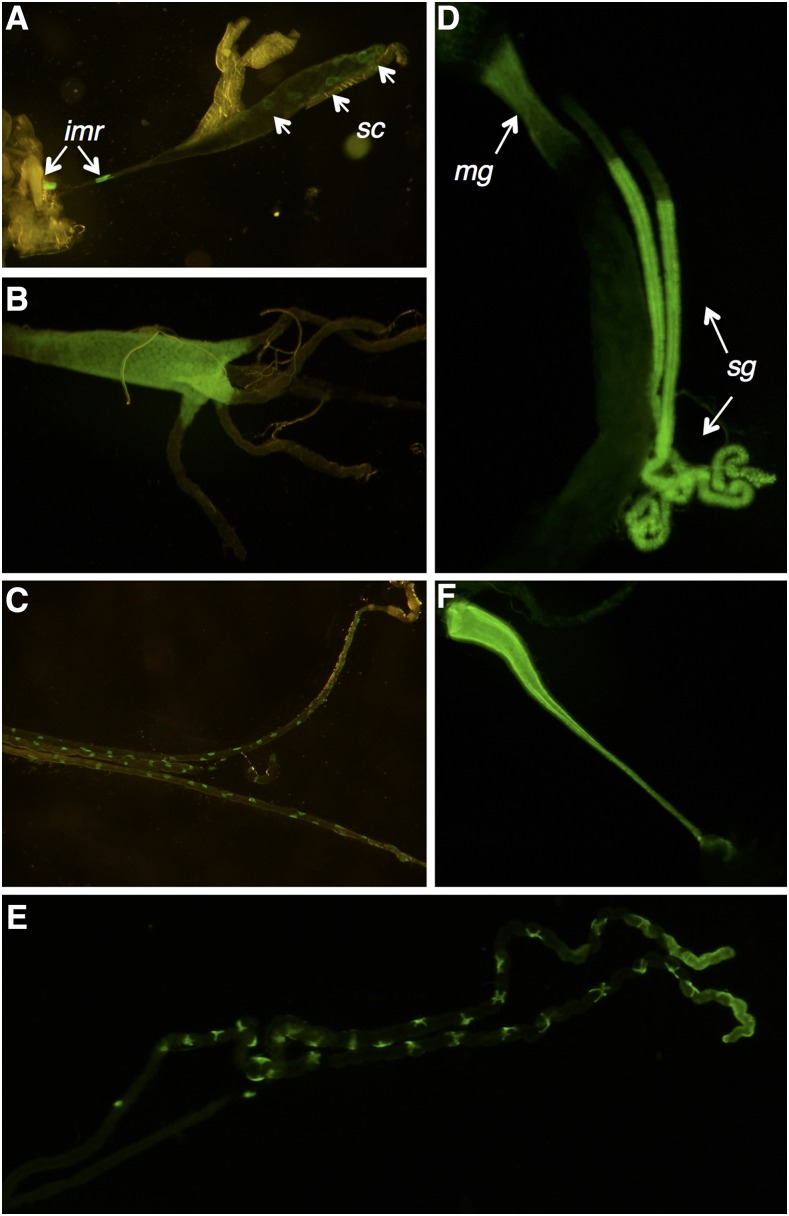
Expression pattern of CG7589 in third instar larvae (A–C) and adults (D–F). Larvae: (A) GFP expression in the larval salivary gland, in secretory cells (sc) and in the imaginal rings (imr), (B) anterior portion of the midgut, and (C) malpighian tubules. Adults: (D) GFP expression in the salivary glands (sg) directly following the salivary duct cells, and the early midgut (mg), (E) the stellate cells of the malpighian tubules, and the (F) male ejaculatory duct. GFP, green fluorescent protein.

The 5′ promoter sequence of CG7589 drove GFP expression in a variety of tissues in larvae and adults (Figure 2). In third instar larvae, GFP patterns indicate that expression of CG7589 occurs in the salivary gland secretory cells and imaginal ring cells, which are precursors of the adult salivary gland (Figure 2A, Curtiss and Heilig 1995). Expression also occurs in the early midgut (section M1–2, Figure 2B) and the malpighian tubules (Figure 2C). In adults, expression occurs in the early midgut and salivary gland (Figure 2D). In the malpighian tubules, expression is present in cells with a star-shaped morphology typical of the stellate cells (Figure 2E, Dow 2009). Expression is also present in the male ejaculatory duct (Figure 2F).

#### CG6927:

**Figure 3:**
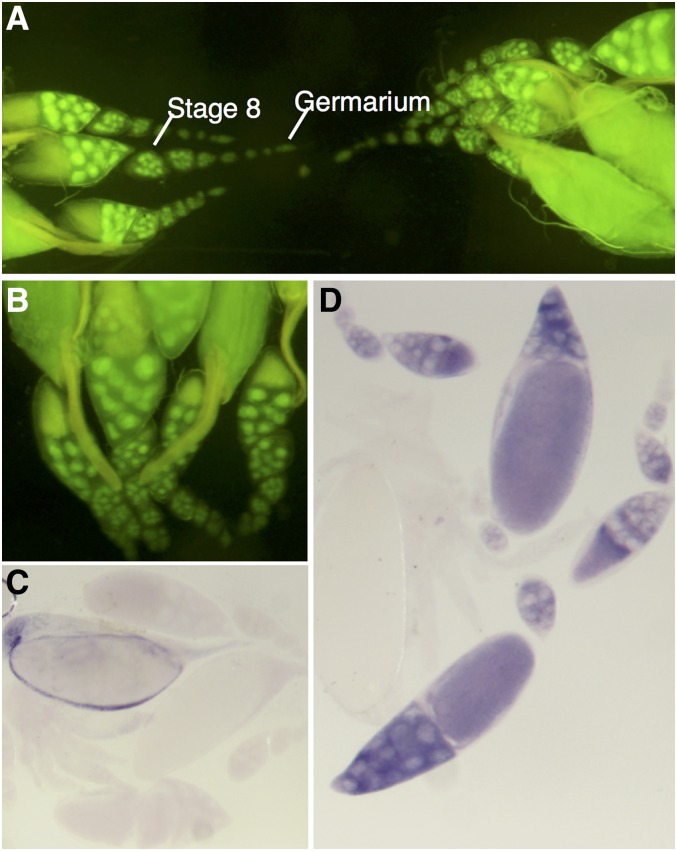
Expression pattern of CG6927. (A and B) Adult female germline. GFP is localized to the nuclei of the nurse cells of oocytes from early on in oocyte development. (C and D) *In situ* hybridization of CG6927 in female reproductive tissue of adult *D. melanogaster*. (C) Negative control using sense probe for CG6927. (D) Positive staining observed in nurse cell cytoplasm using antisense probe for CG6927. GFP, green fluorescent protein.

The 5′promoter sequence of CG6927 in the pStinger construct drove GFP expression throughout the female germline (Figure 3, A–C). Expression is specific to the nurse cells, as seen in the nuclei of the 15 supporting cells contained within the developing cyst. Expression begins early in oogenesis in the anterior germarium, and continues throughout all stages of development of the oocyte. To determine whether the GFP expression seen in the CG6927 5′ promoter constructs accurately reflected the expression of CG6927 in the nurse cells, *in situ* hybridization was carried out using a DIG-labeled, antisense CG6927 RNA probe. Staining was observed in a similar pattern to that seen in the GFP expression, confirming the presence of CG6927 RNA in the cytoplasm of the nurse cells surrounding the developing oocyte (Figure 3D). These expression patterns are consistent with high levels of expression of CG6927 in early embryos (Figure S2B), and genome-wide expression analysis showed enrichment of CG6927 in the oocyte-embryo transition (Kronja *et al.* 2014). CG6927 was also identified to be among a number of proteins phosphorylated in the embryo centrosome (Zhai *et al.* 2008; Habermann *et al.* 2012).

#### CG11340:

**Figure 4:**
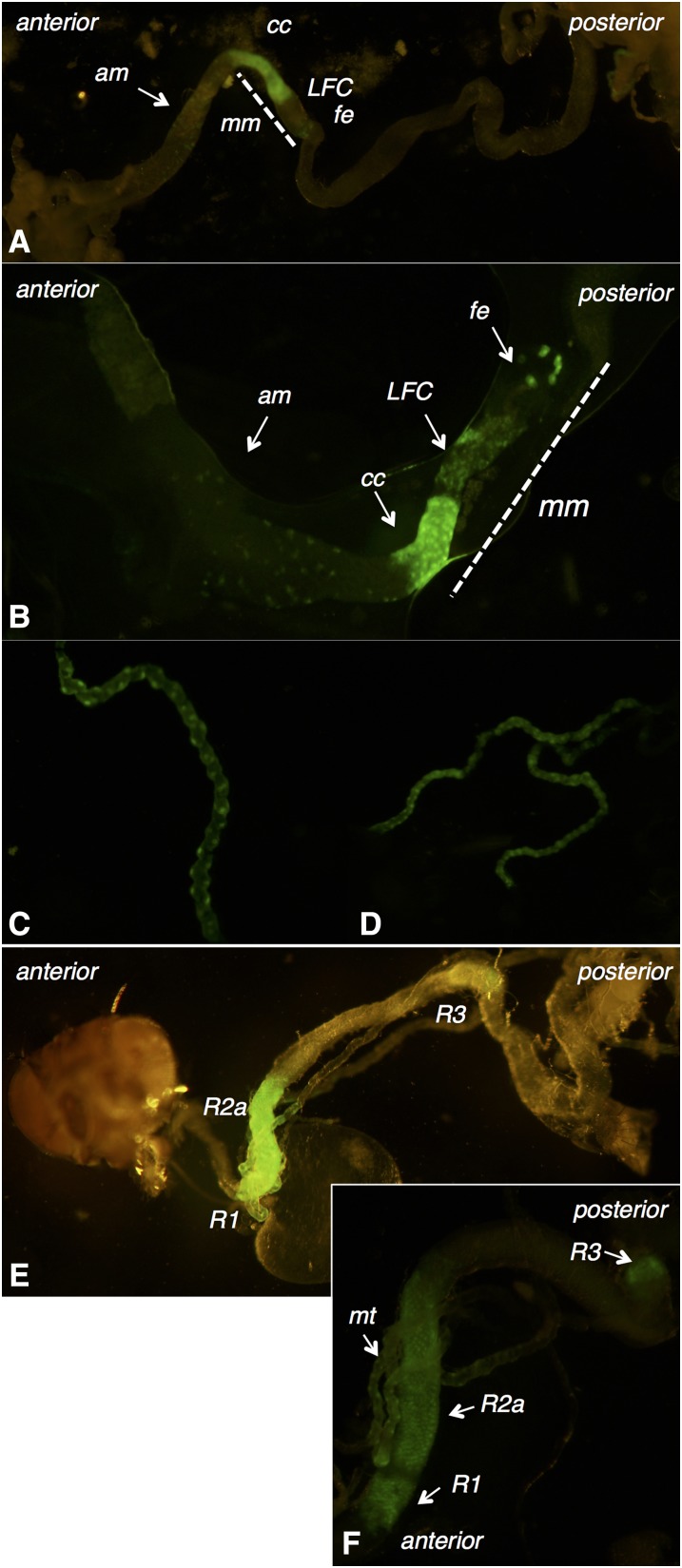
Expression pattern of CG11340. (A and B) The third instar larval midgut, (am- anterior midgut; mm- middle midgut). GFP expression is localized primarily to the middle midgut, divided into cc (copper cells), LFC (large flat cells), and fe (iron cells). (C and D) Malpighian tubule expression in third instar larvae (C) and adults (D). (E and F) The adult midgut. GFP expression is localized to regions R1, R2a, and R3 (50). Also shows malpighian tubules (mt). GFP, green fluorescent protein.

The expression of GFP in individuals containing the CG11340-GAL4-P[acman] construct crossed to nuclear UAS-GFP indicated that CG11340 expression occurs in the malpighian tubules and midgut of third instar larvae (Figure 4, A–C) and adults (Figure 4, D–F). In larvae, midgut expression is most prevalent in the region corresponding to the middle midgut, which contains the copper cells, with lower levels of expression in the large flat cells and iron cells (Filshie *et al.* 1971). Faint expression is also present anterior to the copper cells (Figure 4, A and B). In larvae and adults, malpighian tubule expression appears to be distinct and nonoverlapping with that of CG7589, observed in the principal cells of the tubules (Figure 4, C and D), rather than the stellate cells where CG7589 was present. In the adult midgut, GFP expression was observed in the R2 and R3 compartments of the middle midgut with lower levels of expression in R1, according to the midgut regions determined by Buchon *et al.* (2013) (Figure 4, D and E).

**Figure 5:**
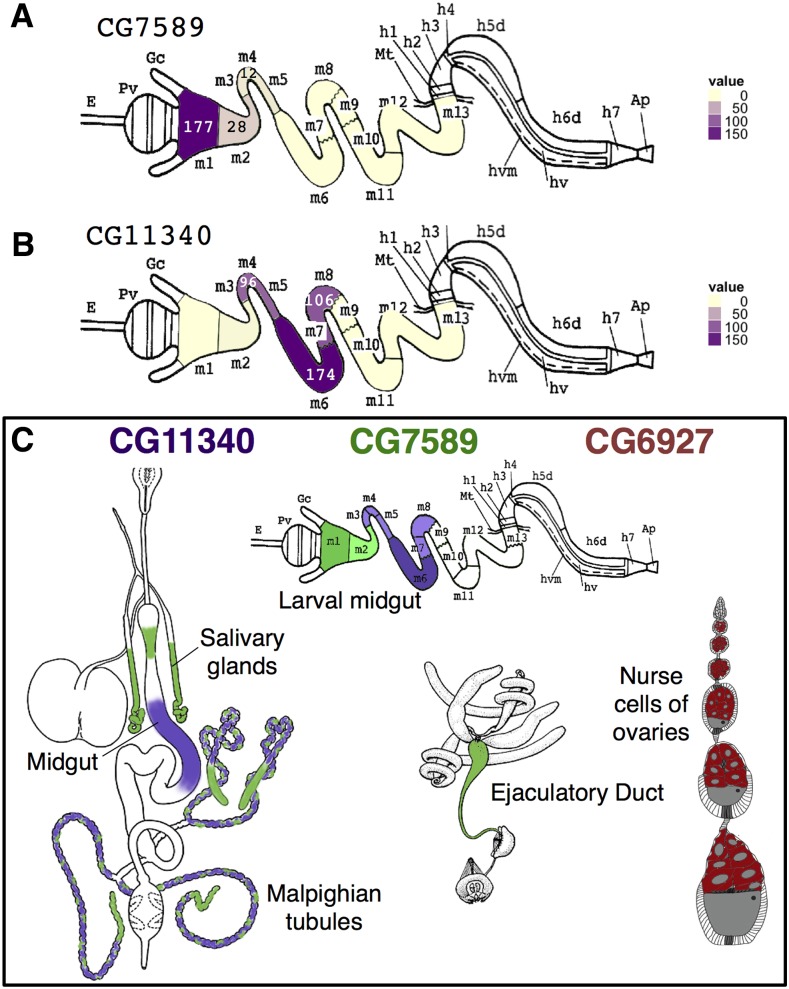
RNA-seq expression levels in third instar larval midgut. (A) CG7589 signal is highest in the M1 region, reducing over the M2–5. (B) CG11340 signal begins in the M3–5 portion and reaches its peak in M6, reducing in M7–8. (C) Summary of gene expression patterns for CG11340 (purple), CG7589 (green), and CG6927 (red), combining promotor-GFP, *in situ* hybridization, and RNA-seq results. Light to dark colored shading represents moderate to high expression levels. GFP, green fluorescent protein. Line drawings adapted from Miller (1950) and Murakami *et al*, (1994).

To confirm the distinct compartmentalized expression of CG7589 and CG11340 in the larval midgut, we examined RNA-transcriptome data obtained from eight specific subsections of the third instar larval midgut (Harrop *et al.* 2014). The larval midgut contains 13 segments, where gene expression can be partitioned into discrete compartments, as identified in enhancer-trap analysis of third instar larval gut tissue (Murakami *et al.* 1994). Expression of CG7589 in the early midgut was confirmed by the high levels of CG7589 RNA in the M1 region, reducing to lower levels in M2 and M3–5 (Figure 5A). CG11340 was detected most highly in the M6 (copper cell) region (Lemaitre and Miguel-Aliaga 2013), with lower levels in the neighboring M3–5 and M7–8 portions (Figure 5B), confirming the GFP expression patterns. Similarly, gene expression in specific regions of the adult midgut have been examined using microarrays (http://flygut.epfl.ch/; Buchon *et al.* 2013). CG11340 was enriched in the R3 region corresponding to the copper cells containing middle midgut. CG7589 was enriched in the R1 region of the early anterior midgut. A summary of the expression of CG7589, CG11340, and CG6927 combining all methods is presented in Figure 5C.

### CG11340 knockout is sensitive to dietary copper

Our data indicate that CG11340 is expressed in the middle midgut of adults and larvae (Figure 4, B–D and Figure 5B), in the same region as the copper cells that function in dietary copper absorption and acid secretion (Filshie *et al.* 1971; Jones *et al.* 2015). A role for chloride in absorption of dietary copper has been previously hypothesized (Lemaitre and Miguel-Aliaga 2013). Thus, we investigated the impact of CG11340 overexpression and knockdown on the mortality of flies exposed to varying levels of dietary copper.

**Figure 6:**
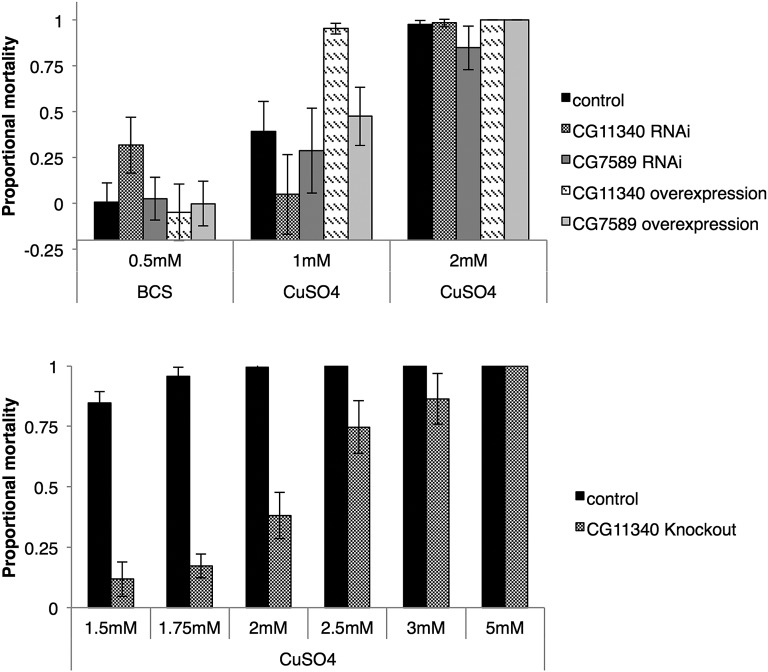
(Top) Overexpression and RNAi knockdown of CG11340 and CG7589 in the midgut, malpighian tubules, and fat body (5′HR driver). Survival on reduced available copper was tested with the addition of 0.5 M of the copper chelator BCS to the media, and increased copper by the addition of 1–2 mM CuSO_4_. Overexpression of CG11340 showed reduced survival on 1 mM CuSO_4_ compared to w^1118^ control and other lines tested, whereas CG11340 knockdown provided increased survival on 1 mM CuSO_4_. (Bottom) Larval to adult mortality of CG11340 knockout and w^1118^ control on 6 doses of CuSO_4_ (1.75–5 mM). Mortality of the CG11340 knockout line is significantly reduced compared to controls (95% C.I. shown). BCS, bathocuproine disulphonate; CuSO_4_, copper (II) sulfate.

We used the 5′HR-GAL4 driver construct to drive overexpression and knockdown of CG11340 in the digestive tract of larvae and adults. The 5′HR-GAL4 driver is a highly potent driver expressing GAL4 throughout the entire midgut, malpighian tubules, and fat body (Chung *et al.* 2007). CG7589 overexpression and knockdown was also tested as a comparison due to the observed expression in neighboring tissues and cells in the early midgut and malpighian tubules (Figure 2 and Figure 5A), alongside a w^1118^ control line. We compared larval to adult mortality of knockdown and overexpression offspring on standard fly media containing either 1 mM CuSO_4_, 2 mM CuSO_4_, 0.5 mM of the copper chelator BCS, or H_2_O as a control. Overexpression of CG11340 showed reduced survival on 1 mM CuSO_4_ compared to w^1118^ control and all other lines (Figure 6, top). In contrast, CG11340 knockdown provided increased survival on 1 mM CuSO_4_. CG7589 overexpression showed no significant differences from w^1118^ survival on BCS or CuSO_4_-supplemented media.

To confirm the copper tolerance observed with CG11340 knockdown, we generated a knockout of the CG11340 gene using ends-out targeting (Gong and Golic 2003; Huang *et al.* 2008). Knockout was confirmed by southern blot (Figure S5), and absence of the CG11340 transcript was confirmed with RT-PCR (data not shown). Knockout flies showed no observable signs of reduced viability under standard rearing conditions. We compared larval to adult mortality of CG11340 knockout and w^1118^ controls on six doses of CuSO_4_ (1.75–5 mM, Figure 6, bottom). Mortality of the CG11340 knockout line was significantly reduced compared to controls (Figure 6, bottom), confirming the copper tolerance phenotype. Similar copper tolerance was observed when CuSO_4_ was substituted for CuCl_2_, while no tolerance to a second metal, zinc, was observed when knockout flies were placed on media containing 5–10 mM of either ZnCl_2_ or ZnSO_4_ (data not shown), indicating that the phenotype was specific to copper.

## Discussion

The Insect Group I LGCCs are among the most divergent subunits in the LGIC superfamily. The multiple copies present in *A. pisum* and *T. castaneum* are monophyletic, suggesting independent duplication events occurred during the evolutionary history of the Coleoptera and Hemiptera Orders. In *T. castaneum*, the three paralogs are arranged tandemly within 10 kb on the same linkage group that most likely arose by tandem duplication (Figure S3, Jones and Sattelle 2007). Similarly, in the ant *Acr. echinator*, the two subunits are tandemly arranged on the same scaffold (Figure S3) and most likely arose from a recent duplication event, as investigation of other ant genomes indicated that only one ortholog was present. In the Dipteran lineage, duplication to generate the three paralogous Insect Group I subunits CG6927, CG7589, and CG11340 occurred after divergence of Culicimorpha, with the observation of single-copy mosquitoes and multi-copy Muscomorpha. The absence of CG11340 in the Tsetse fly *G. mortisans* suggests that the three subunits arose via sequential duplications, with CG11340 emerging as the most recent subunit. Alternatively, the CG11340 subunit may have been lost from *G. mortisans*, or be present in a gap in the sequenced genomic assembly for this species. However, the latter is unlikely owing to the high quality genome assembly estimated to be 99% complete (International Glossina Genome Initiative 2014). The presence of multiple intron-exon boundaries is common between single-ortholog insects, and *D. melanogaster* CG7589 orthologs confirm that the ancestral subunit in Diptera is CG7589, and that CG6927 and CG11340 arose from duplication events. The presence of a single intron in each of CG6927 and CG11340, and the remote genomic locations relative to CG7589 (CG6927, X chromosome; CG11340, 3R; and CG7589 3L, Figure S3) indicate that the mode of duplication was retrotransposition, in contrast to the tandemly duplicated subunits in *T. castaneum* and *Acr. echinator*, representing different mechanisms of gene duplication between different insect lineages.

Divergent copy number throughout insect evolution raises questions about the functions of the subunits in this branch of the LGCC family; however, the Insect Group I subunits have previously remained uncharacterized. Thus, our delineation of the discrete cell type-specific, nonneuronal expression of the three *D. melanogaster* subunits, CG7589, CG6927, and CG11340, has major evolutionary and functional implications.

The three *D. melanogaster* Insect Group I LGCC paralogs are a quintessential example of one of the possible evolutionary trajectories of gene duplications. Duplicate genes are generally purged from the genome via pseudogenization, unless they provide a selective advantage and acquire a specific essential or beneficial role (Levasseur and Pontarotti 2011). Duplications can arise by multiple mechanisms. The distant locations of the *D. melanogaster* Insect Group I subunits (X, 3L, and 3R chromosomes; Jones and Sattelle 2007), and the lack of introns in duplicate copies, indicates that CG6927 and CG11340 duplicates are ancient functional retrogenes (Kaessmann *et al.* 2009) arising via retrotransposition of mRNA of the ancestral subunit CG7589 in Diptera after the Culicimorpha and Muscoporpha split (Figure 1 and Figure S3). The divergent expression patterns indicate that CG6927 (ovaries; Figure 3 and Figure 5C) and CG11340 (copper cells of the midgut, primary cells of malpighian tubules; Figure 4 and Figure 5C) have taken on distinct roles from their progenitor locus CG7589 (salivary glands, anterior midgut, stellate cells of malpighian tubules, and ejaculatory duct; Figure 2 and Figure 5C). This scenario aligns with theoretical fates of genes postduplication, whereby duplicate copies are maintained either because they acquire novel expression patterns (neofunctionalization), or because they assume one aspect of the previous expression pattern of their parental gene (subfunctionalization; Levasseur and Pontarotti 2011). The nature of how functional retrogenes recruit regulatory elements is currently unclear in *D. melanogaster*, though may be due to accompanying 5′ UTRs, or modification of preexisting upstream sequences present in insertion sites (Kaessmann *et al.* 2009).

We report a novel, nonneuronal role for a LGCC in insects. Based on the expression of CG11340 in the copper cells of the larval and adult midgut (Figure 4 and Figure 5), we surmise that the function of CG11340 is linked to copper. We have shown that loss of CG11340 expression results in copper tolerance, and overexpression causes a copper sensitive phenotype (Figure 6). The copper cell region of the middle midgut is responsible for acid secretion and maintains a pH < 4.0 (Shanbhag and Tripathi 2009). Copper feeding inhibits acid secretion (McNulty *et al.* 2001). Recently, CG11340 was shown to form a homomeric pH-sensitive chloride channel (Feingold 2014). Two other LGICs, *D. melanogaster pHCl* and *C. elegans* PBO-5/6, are gated by protons and sensitive to pH (Schnizler *et al.* 2005; Beg *et al.* 2008). CG11340 channels were shown to be inactive at an acidic pH, with activation occurring with increasing alkalinity (Feingold 2014). CG11340 could therefore maintain gut pH by facilitating chloride influx into the copper cells of the middle midgut via CG11340 when pH increases. Loss of CG11340 could result in reduced gut acidity, subsequently reducing copper uptake and enabling higher tolerance to dietary copper (Figure 6). The role of CG11340 in the malpighian tubules may also be linked to the copper phenotype and compound the effects seen in both the overexpression and deletion lines. The role of both CG7589 and CG11340 in the malpighian tubules has been investigated and these subunits are important in mediating the response to osmotic stress (Feingold 2014). CG11340 is also expressed in innervated regions of the adult midgut that express choline acyltransferase (Buchon *et al.* 2013), however, Feingold (2014) ruled out acetylcholine as an activating ligand of CG11340. An alternative role for CG11340 could therefore be in the midgut neuroendocrine system.

Overexpression and RNAi of CG7589 was also tested on copper (Figure 6, top) and did not show any copper-related phenotype. This suggests a specific role for the CG11340 subunit in this process, and further illustrates a functional divergence of these two subunit paralogs and their relative midgut compartmentalization (Figure 5).

The Insect Group I subunits are three rare examples of Cys-loop LGICs that function outside of neurotransmission. The *PBO-5* and *-6* subunits in *C. elegans* are two other LGIC subunits that have a defined role outside of neurotransmission. These subunits are gated by protons, and the resulting cation influx is involved in muscle contraction (Beg *et al.* 2008). Eukaryotic LGICs exhibit remarkable structural homology with prokaryotic homologs (Corringer *et al.* 2012), suggesting that the basic subunit structure predates the evolution of the nervous system. This suggests that the basic role of LGICs in ion transport has been adopted by the nervous system for fast synaptic signaling in vertebrates and invertebrates. The function of the Insect Group I subunits as chloride channels in secretory tissues may more closely resemble the ancestral functions of their homologous prokaryotic chemoreceptors (Tasneem *et al.* 2004). In line with this hypothesis, a proton-gated prokaryotic LGIC has been characterized, demonstrating a conserved functional origin of the LGIC family (Bocquet *et al.* 2007).

LGCCs continue to be useful target sites for insecticides (Raymond and Sattelle 2002), including novel chemical classes with distinct modes of action (Weber and Selzer 2016). However, if target subunits are highly conserved, it is more likely that unwanted effects will be seen in nontarget, beneficial organisms. Characterization of nematode-specific chloride channels in *C. elegans* has illustrated that subunit divergence can be useful for designing new chemicals (Wolstenholme 2012; Beech and Neveu 2015; Wever *et al.* 2015). Thus, a full understanding of the functional repertoire of all insect chloride channel subunits may allow for the rational design of insecticide targets that are pest-specific in species where divergent subunits have been identified. A key element to rational insecticide design is an understanding of the functional redundancy of a target and whether loss of function variants are viable (Perry *et al.* 2011). We have shown that a CG11340 knockout is viable. Therefore, in this case, the CG11340 subunit would not offer a promising insecticide target: loss-of-function mutants could easily generate resistance. In contrast, CG7589 shows dramatically reduced viability when transcript levels are reduced by RNAi (Figure S6), with individuals showing lethality at d 3–4. Similarly, *P*-element insertion mutants that abolish CG7589 expression exhibit lethality (Feingold 2014). Observations of lethality when subunit expression is reduced or absent are equivalent to the phenotype that would be observed if the subunit was blocked by a chemical insecticide. Therefore, investigating knockout and/or knockdown mutants for lethal phenotypes could provide promising insecticide target sites.

## Supplementary Material

Supplemental Material
