# Supplementary material for: Evolution, Expression, and Function of Nonneuronal Ligand-Gated Chloride Channels in Drosophila melanogaster
Source: G3 (Bethesda). 2016 May 4;6(7):2003–12. doi: 10.1534/g3.116.029546 (PMC4938653; doi:10.1534/g3.116.029546)
Supplement: Supplemental Material [file supp_6_7_2003__index.html]

Evolution, Expression, and Function of Nonneuronal Ligand-Gated Chloride Channels in Drosophila melanogaster — Supplemental Material 

# Evolution, Expression, and Function of Nonneuronal Ligand-Gated Chloride Channels in *Drosophila melanogaster*

## Supplemental Material for Remnant *et al.*, 2016

**Files in this Data Supplement:**

- Figure S1 - LGCC gene copy number present in the genome sequences of insect species. (.pdf, 261 KB)
- Figure S2 - FlyAtlas spatial expression information for the 12 *Drosophila melanogaster* LGCC subunits. (.pdf, 597 KB)
- Figure S3 - Genomic organisation of Insect group I subunits. (.pdf, 485 KB)
- Figure S4 - Construction of 5'upstream promoter constructs. (.pdf, 180 KB)
- Figure S5 - Southern Blot of parental (white) and CG11340 knockout (line 4A2) using a 3kb homology arm probe. (.pdf, 1002 KB)
- Figure S6 - Tubulin-GAL4 driver over TM3 balancer crossed to RNAi hairpin lines for CG11340, CG6927 and CG7589 (TRiP (A); and KK (B)), and non-hairpin control. (.pdf, 181 KB)
- File S1 - Sequences used in the LGCC Insect Group 1 alignment. (.zip, 13 KB)
