# Supplementary material for: Evolution, Expression, and Function of Nonneuronal Ligand-Gated Chloride Channels in Drosophila melanogaster
Source: G3 (Bethesda). 2016 May 4;6(7):2003–12. doi: 10.1534/g3.116.029546 (PMC4938653; doi:10.1534/g3.116.029546)
Supplement: Supplemental Material [file supp_g3.116.029546_FigureS1.pdf]

Figure S1

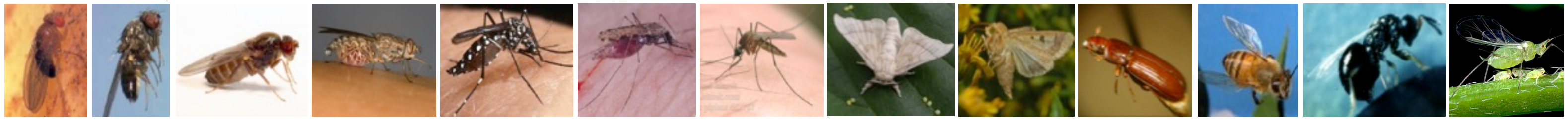

| D. melanogaster | D. pseudoobscura | D. virilis | G. mortisans | A. aegypti | A. gambiae | C. pipens | B. mori | H. armigera | T. castaneum | A. mellifera | N. vitripennis | A. pisum |
|---|---|---|---|---|---|---|---|---|---|---|---|---|
| **12** | **12** +1ψ | **12** | **11** | **10** | **10** | **10** | **12** | **12** | **12** | **10** | **10** | **10** |
| **GluClα** | 1 | 1 | 1 | 1 | 1 | 1 | 1 | 1 | 1 | 1 | 1 | 1 |
| **Rdl** | 1 | 1 | 1 | 1 | 1 | 1 | **[a]3** | **3** | 1 | 1 | 1 | **2** |
| **Lcch3** | 1 | 1 | 1 | 1 | 1 | 1 | 1 | 1 | 1 | 1 | 1 | - |
| **CG8916** | 1 | 1 | 1 | 1 | 1 | 1 | 1 | 1 | 1 | 1 | 1 | - |
| **Grd** | 1 | 1 | 1 | 1 | 1 | 1 | 1 | 1 | 1 | 1 | 1 | - |
| **CG12344** | 1 | 1 | 1 | 1 | 1 | 1 | 1 | 1 | 1 | 1 | 1 | - |
| **ort** | 1 | 1 | 1 | 1 | 1 | 1 | 1 | 1 | 1 | 1 | 1 | - |
| **HisCl1** | 1 | 1 | 1 | 1 | 1 | 1 | 1 | 1 | 1 | 1 | 1 | 1 |
| **pHCl** | 1 | 1 | 1 | 1 | 1 | 1 | 1 | 1 | 1 | 1 | 1 | **2** |
| **CG11340** | **1** | **1** | - | - | - | - | - | - | - | - | - | **1** |
| **CG7589** | **1** | **1** | **1** | - | - | - | **1** | **1** | - | - | - | **1** |
| **CG6927** | **2 (1ψ)** | **1** | **1** | **1** | **1** | **1** | - | - | **3** | **1** | **1** | - |
| *other* | - | - | - | - | - | - | - | - | - | - | - | *2* |

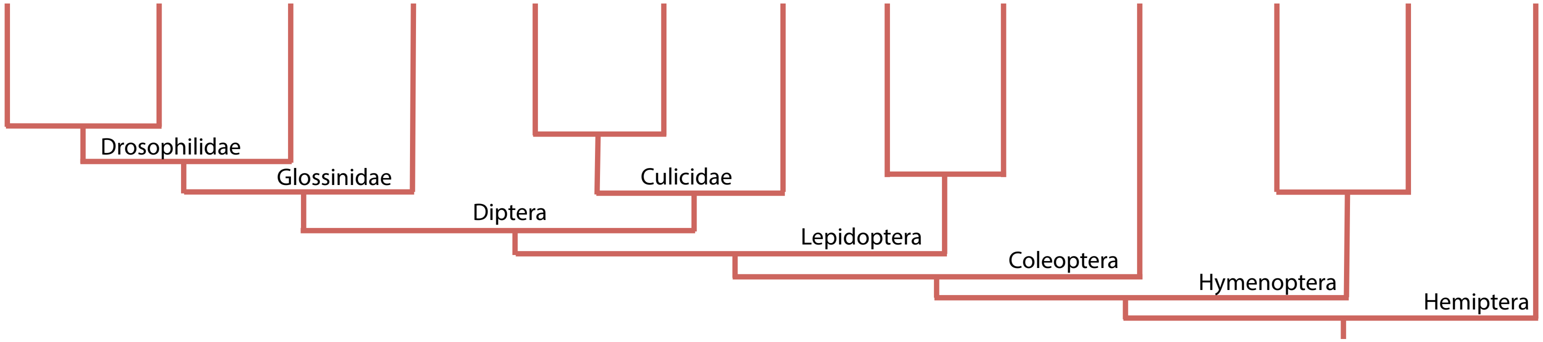


**Figure S1.** LGCC gene copy number present in the genome sequences of insect species. Total number of subunits in each species is indicated at the top of the figure. Orthologues are compared to the *D. melanogaster* reference gene set. Copy number was determined by BLAST searches.
