# Supplementary material for: Evolution, Expression, and Function of Nonneuronal Ligand-Gated Chloride Channels in Drosophila melanogaster
Source: G3 (Bethesda). 2016 May 4;6(7):2003–12. doi: 10.1534/g3.116.029546 (PMC4938653; doi:10.1534/g3.116.029546)
Supplement: Supplemental Material [file supp_g3.116.029546_FigureS2.pdf]

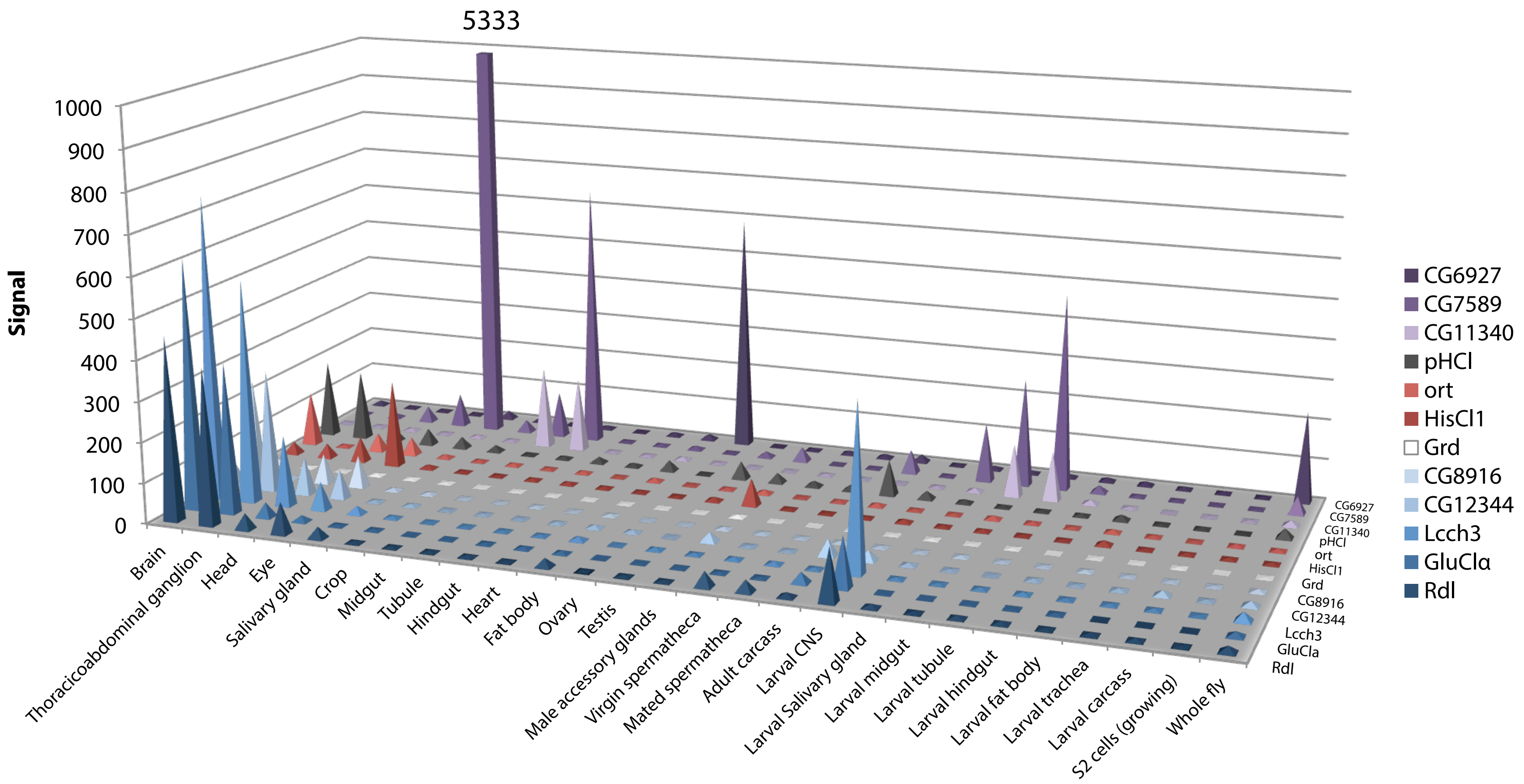

Figure S2 a. FlyAtlas spatial expression information for the 12 *Drosophila melanogaster* LGCC subunits.

Figure S2 b

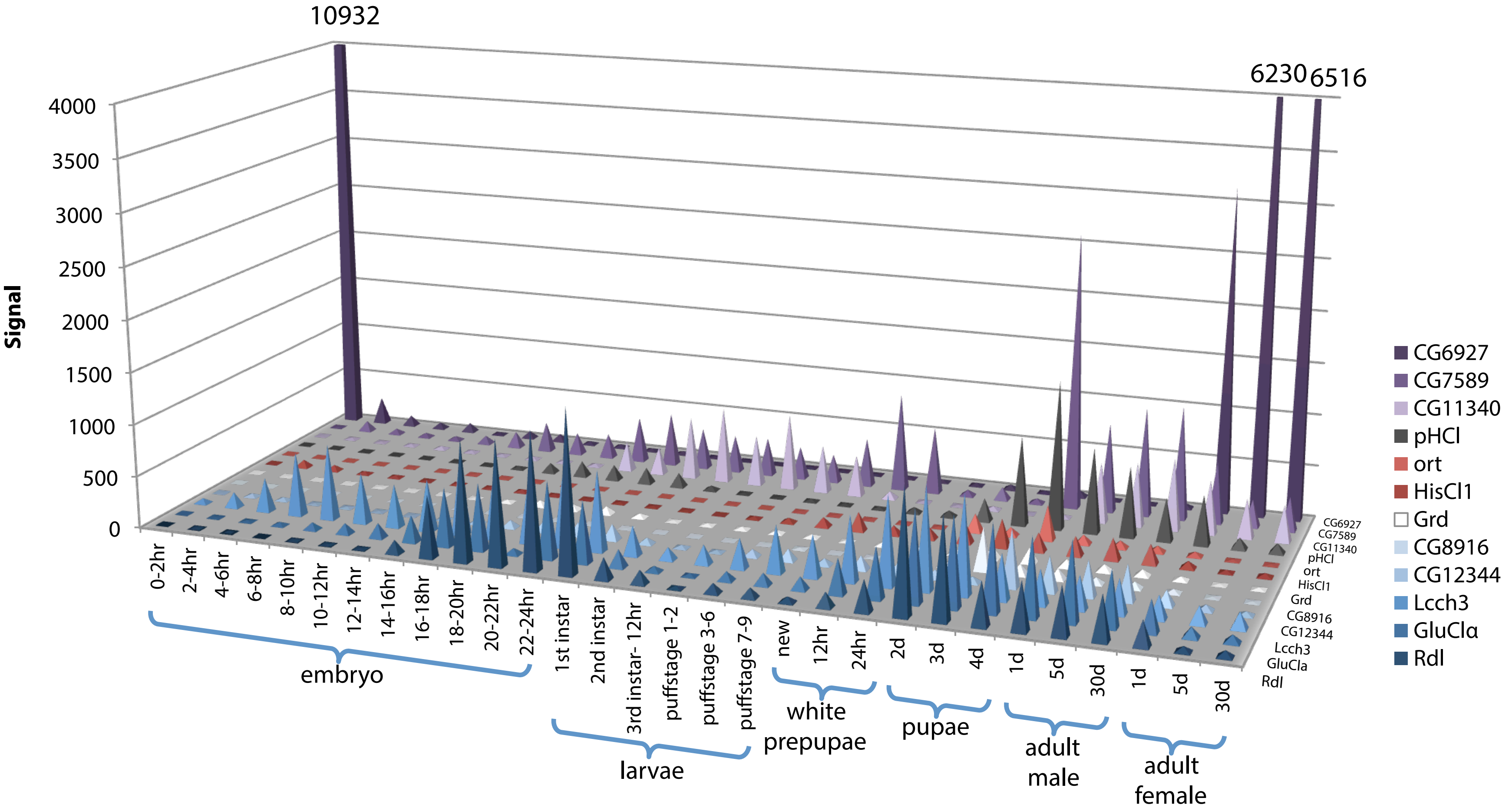

Figure S2 b ModEncode temporal expression information for the 12 *Drosophila melanogaster* LGCC subunits.
