# Supplementary material for: Evolution, Expression, and Function of Nonneuronal Ligand-Gated Chloride Channels in Drosophila melanogaster
Source: G3 (Bethesda). 2016 May 4;6(7):2003–12. doi: 10.1534/g3.116.029546 (PMC4938653; doi:10.1534/g3.116.029546)
Supplement: Supplemental Material [file supp_g3.116.029546_FigureS3.pdf]

Figure S3

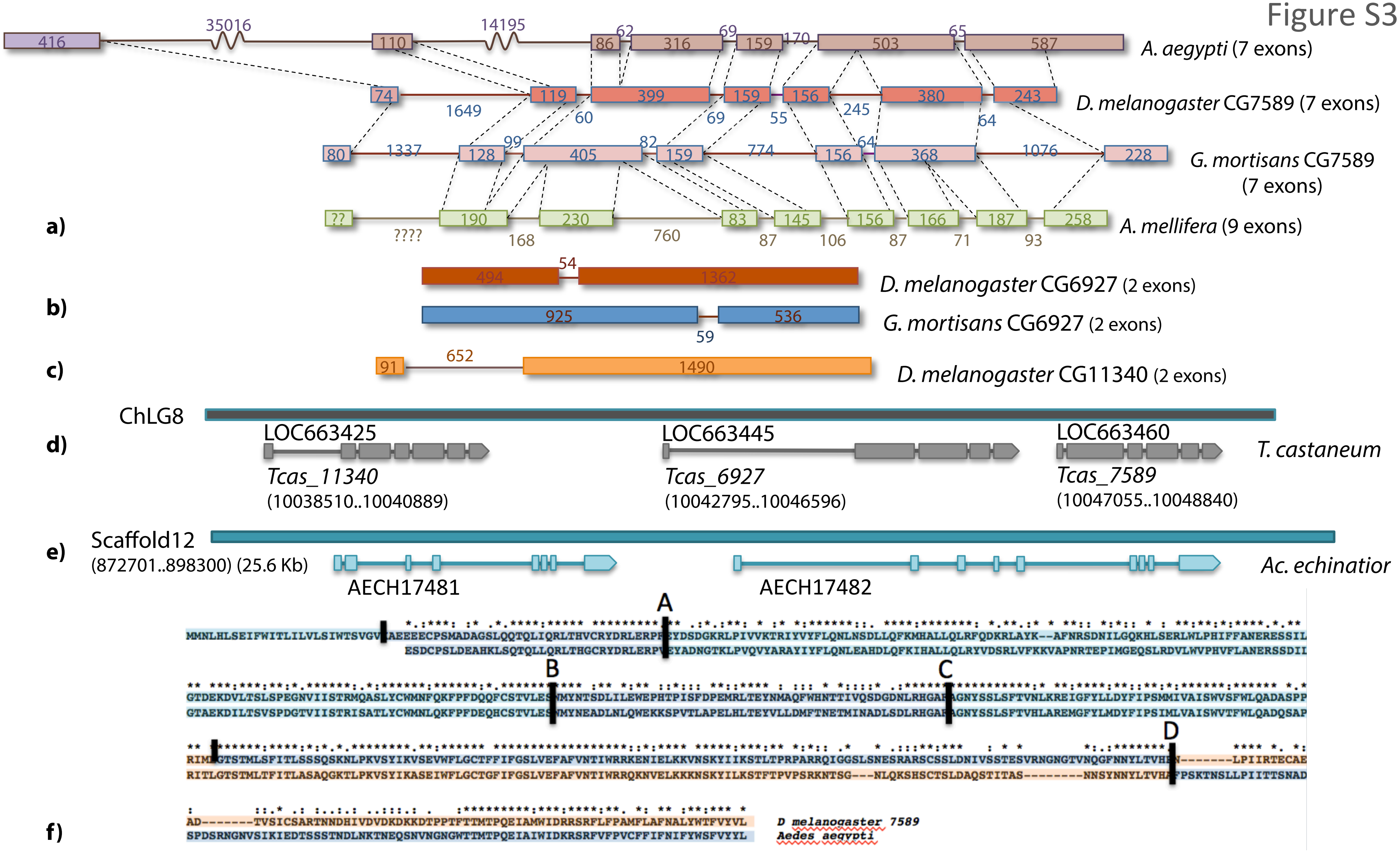

Figure S3. Genomic organisation of Insect group I subunits. **a)** Exon structure of CG7589 in *Aedes aegypti, Drosophila melanogaster, Glossinia morisans* and *Apis mellifera,* showing corresponding intron-exon boundries. **b)** CG6927 in *D. melanogaster* and *G. mortisans* contain a single intron at different sites in the coding region. **c)** CG11340 from *D.* melanogaster contains a single intron early in the coding region. **d)** The *Tribolium castaneum* trio are tandemly arranged on the same linkage group and contain a similar number of exons. **e)** The Acromyrmex echinatior duo are tandemly arranged with a similar number of exons. **f)** Detailed comparison of CG7589 amino acid alignment of *D. melanogaster* and *Aedes aegypti*, with position of introns in common indicated by A-D
