# Supplementary material for: Evolution, Expression, and Function of Nonneuronal Ligand-Gated Chloride Channels in Drosophila melanogaster
Source: G3 (Bethesda). 2016 May 4;6(7):2003–12. doi: 10.1534/g3.116.029546 (PMC4938653; doi:10.1534/g3.116.029546)
Supplement: Supplemental Material [file supp_g3.116.029546_FigureS4.pdf]

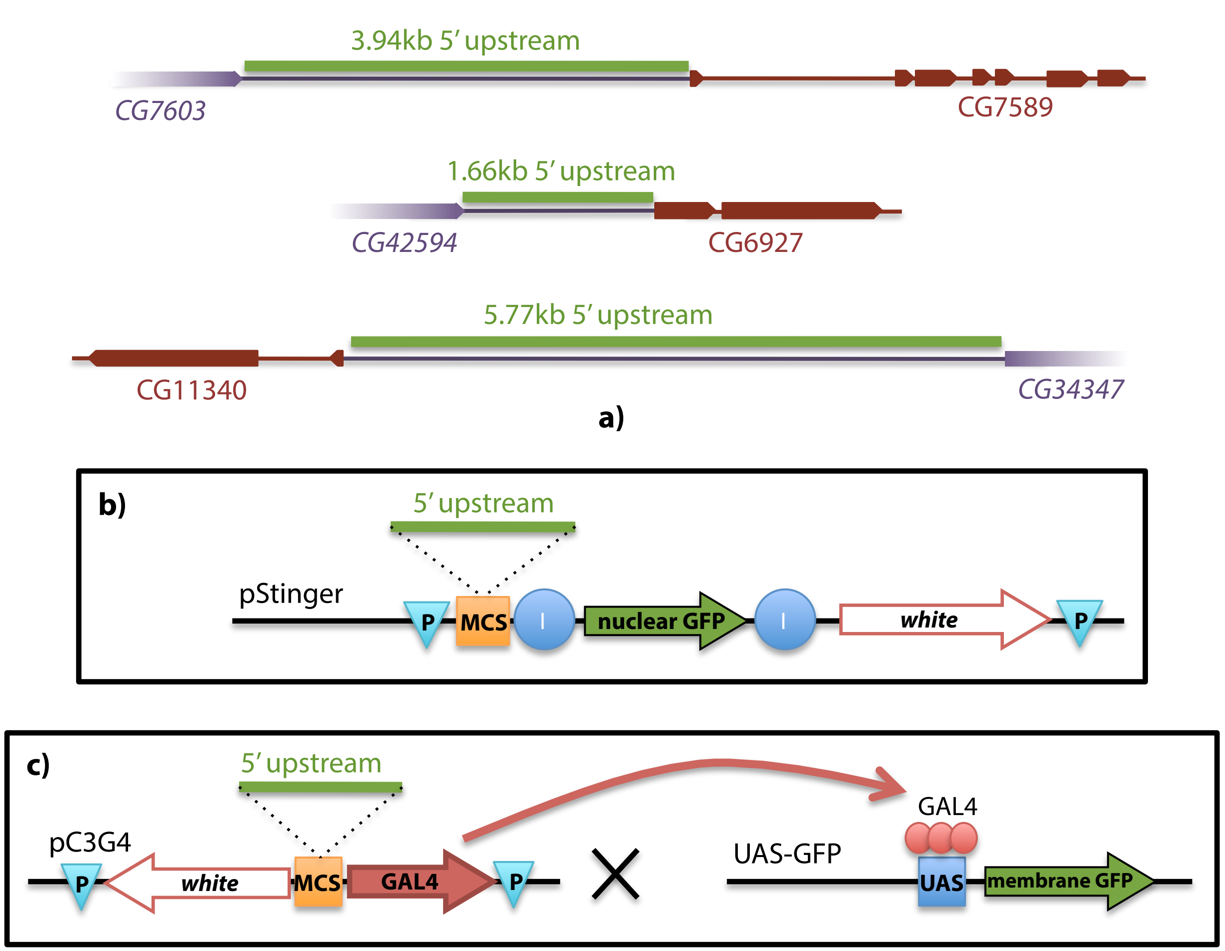

Figure S4. Construction of 5'upstream promoter constructs. **a)** 5' upstream sequence of CG7589 (3.94kb), CG6927 (1.66kb) and CG11340 (5.04kb). **b)** The pStinger vector (Barolo *et al.*, 2000), showing position of nuclear GFP downstream from the multi-cloning site (MCS), where the 5' upstream regions are inserted. **c)** pC3G4 vector (derived from Thummel and Pirotta, 1991, and Brand and Perrimon, 1993) allows the direct insertion of identified promoter fragments in front of a Gal4 sequence. This line can be crossed to a UAS-contaning line, such as UAS-mGFP, and the GAL4 drive expression of GFP by binding to the UAS sequence.
