# Supplementary material for: Evolution, Expression, and Function of Nonneuronal Ligand-Gated Chloride Channels in Drosophila melanogaster
Source: G3 (Bethesda). 2016 May 4;6(7):2003–12. doi: 10.1534/g3.116.029546 (PMC4938653; doi:10.1534/g3.116.029546)
Supplement: Supplemental Material [file supp_g3.116.029546_FigureS5.pdf]

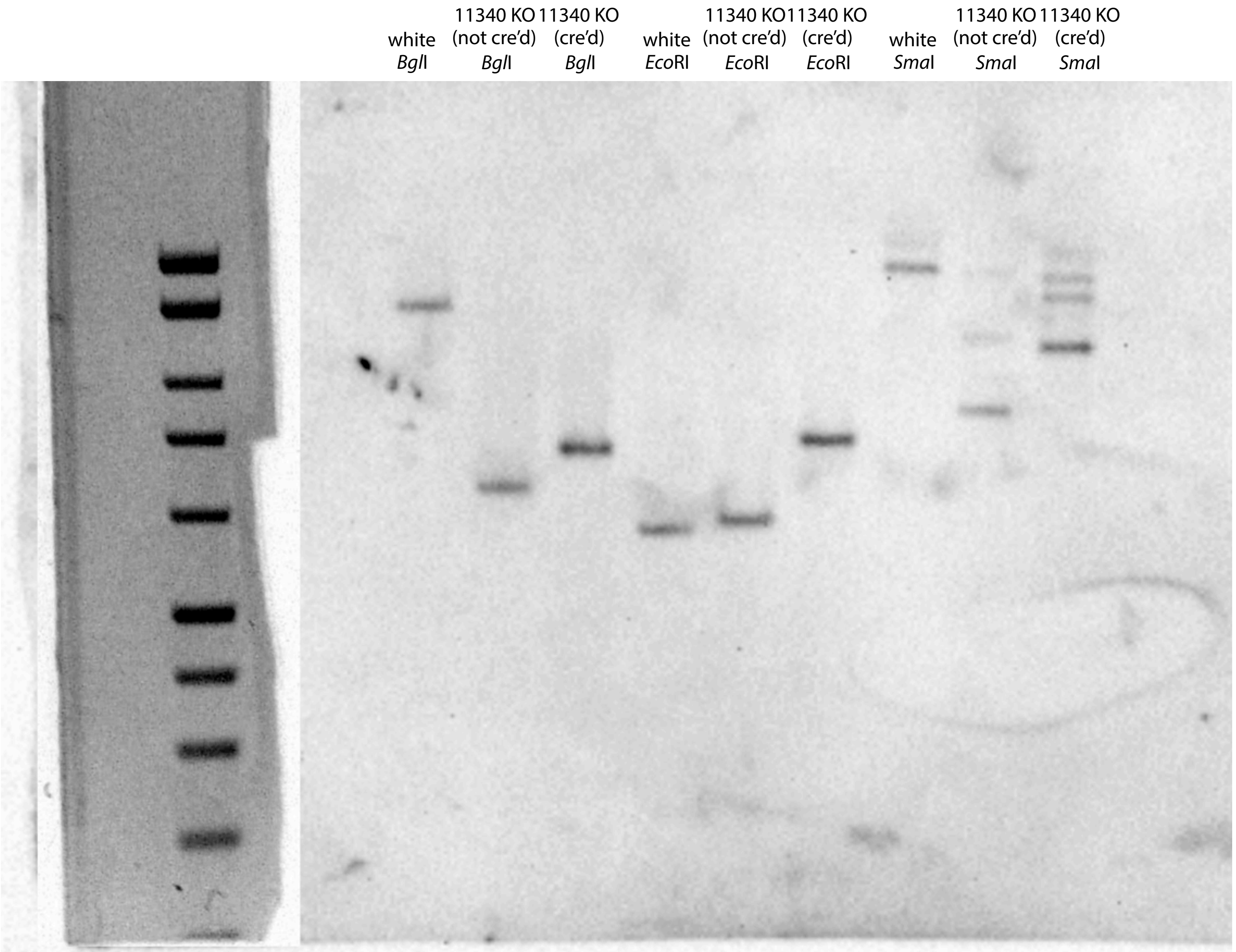

Figure S5. Southern Blot of parental (white) and CG11340 knockout (line 4A2) using a 3kb homology arm probe. Image is of a 5 minute exposure. 1kb ladder (lane 1 - Bioline). Genomic DNA from parental, 11340 KO and 11340 cre-lox KO blotted using DNA digested with *Bgl*I (lanes 2-4), *Eco*RV (lanes 5-7) and *Sma*I (lanes 8-10). Note: sequencing across the deletion and probe region revealed an additional EcoRV site present in the parental strain that led to the smaller than expected size of the knockout and this enzyme was not used to verify other deletion lines.
