# Supplementary material for: Evolution, Expression, and Function of Nonneuronal Ligand-Gated Chloride Channels in Drosophila melanogaster
Source: G3 (Bethesda). 2016 May 4;6(7):2003–12. doi: 10.1534/g3.116.029546 (PMC4938653; doi:10.1534/g3.116.029546)
Supplement: Supplemental Material [file supp_g3.116.029546_FigureS6.pdf]

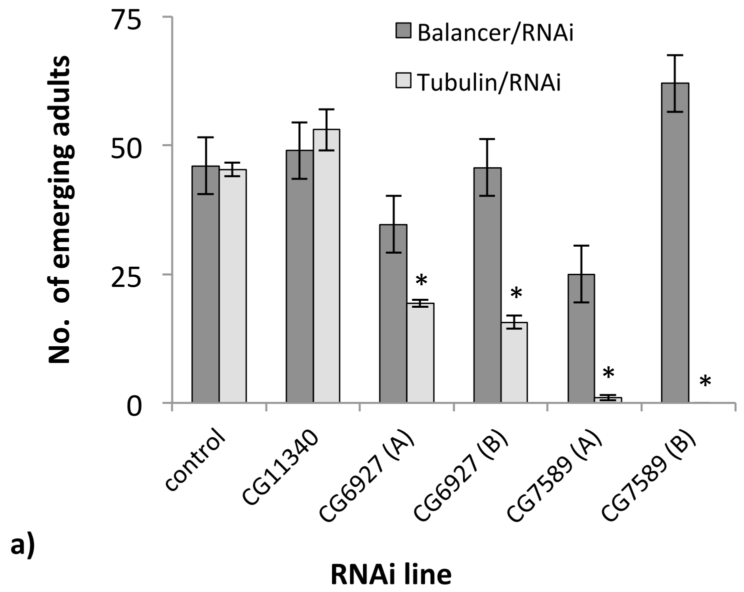

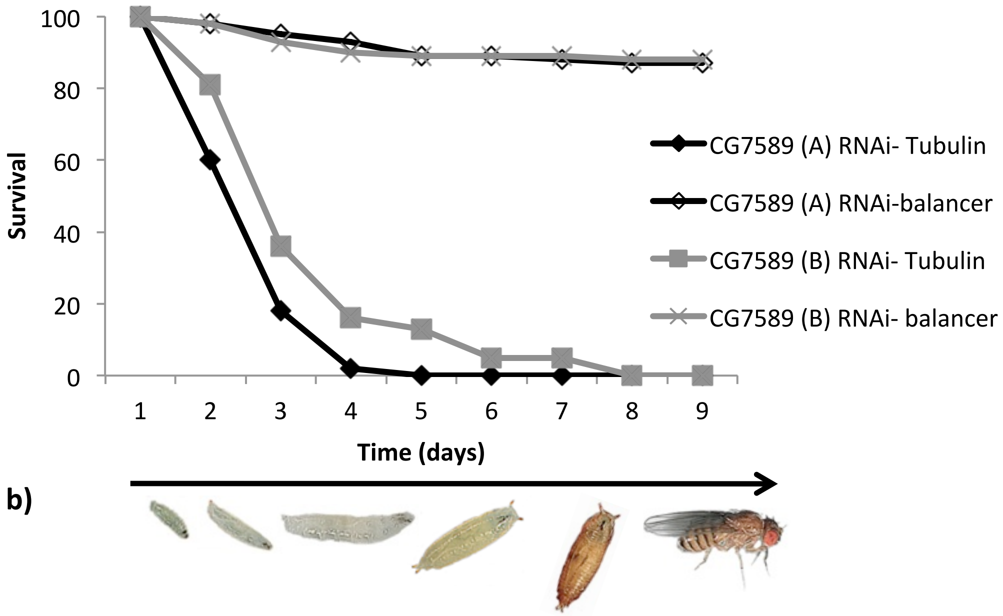

Figure S6. a) Tubulin-GAL4 driver over TM3 balancer crossed to RNAi hairpin lines for CG11340, CG6927 and CG7589 (TRiP (A); and KK (B)), and non-hairpin control. Number of balancer to non-balancer emerging adults were compared to determine if RNAi-mediated knockdown results in reduced viability. CG11340 tubulin-RNAi showed equivalent viability compared to balancers, while both CG6927 showed reduced viability. **b)** *Tubulin* RNAi of CG7589 examined over 9 days during development to determine lethal time point. Y axis indicates percent survival. CG7589 RNAi offspring survival compared compared to GFP balancer controls.
